# Effects of the moderate stress exposure on the short-term memory capacity: An experimental study in fire cadets

**DOI:** 10.1192/j.eurpsy.2021.2064

**Published:** 2021-08-13

**Authors:** E. Bityutskaya, N. Lebedeva, Y. Tsalikova

**Affiliations:** 1 Faculty Of Psychology, Lomonosov Moscow State University, Moscow, Russian Federation; 2 Diagnostics Department, Moscow Metropolitan Governance University, Moscow, Russian Federation

**Keywords:** memory capacity, short-term memory, systolic wave amplitude, stress

## Abstract

**Introduction:**

Future firefighters are selected and trained to perform well under pressure and stress.

**Objectives:**

The research is focused on the experimental study of fire cadets’ memory capacity under stress.

**Methods:**

The study follows Solomon Four Group Design with two variables: stress stimuli (exposure/non-exposure), fire cadets (n=50)/civilian students (n=40). Two series of The Digit Span Test measurements (DST) were performed. Heart rate, EMG, systolic wave amplitude, pulse transit time were measured during the experiment to determine the respondents’ stress levels.

**Results:**

Memory capacity in fire cadets under stress (n=30) significantly increased (Wilcoxon match-pairs rank test, р = 0.001; 1st DST series, neutral stimuli: M=6.53, SE=0.17, SD=0.96; 2st DST series, stressful stimuli: M=7.3, SE=0.21, SD=1.16), the obtained effect size was medium (Cohen’s d = 0.7232). There was no significant change in memory capacity in civilian students under stress (n=20, Wilcoxon test, р=0.452; 1st DST series, neutral stimuli: M=6.78, SE=0.23, SD=1.02; 2st DST series, stressful stimuli: M=6.7, SE=0.23, SD=1.04). Moreover, there was no significant change in memory capacity in fire cadets that were not under stress (n=20, Wilcoxon test, р = 0.628; 1st DST series, neutral stimuli: M=6.88, SE=0.16, SD=0.70; 2st DST series, neutral stimuli: M=6.78, SE=0.16, SD=0.73). Systolic wave amplitude in the stress-exposed groups changed more pronouncedly in students (Mann–Whitney test, z=-2.131; p = 0.033) compared to cadets.

**Conclusions:**

In most of the fire cadets, moderate stress exposure resulted in a memory capacity increase.

**Disclosure:**

No significant relationships.

